# Improving Prediction of Falls and Cognitive Impairment in Parkinson Disease: Protocol for a Decentralized Observational Study

**DOI:** 10.2196/71955

**Published:** 2025-08-27

**Authors:** Audrey Hoyt, Casey Dorney, Peggy Auinger, Kathryn Murphy, Michelle Porto, Katrina Schmier, Renee Wilson, James C Beck, Stephanie Benvengo, Kevin Biglan, E Ray Dorsey, Alberto J Espay, Eric A Macklin, Mariana HG Monje, Dan Novak, David Oakes, Larsson Omberg, Michael A Schwarzschild, Solveig K Sieberts, Tanya Simuni, Caroline M Tanner, Daniel Weintraub, Ruth B Schneider

**Affiliations:** 1 University of Rochester Medical Center Rochester United States; 2 Parkinson's Foundation New York, NY United States; 3 Michael J. Fox Foundation New York United States; 4 Eli Lilly (United States) Indianapolis United States; 5 University of Cincinnati Medical Center Cincinnati United States; 6 Massachusetts General Hospital Boston, MA United States; 7 Northwestern University Chicago, IL United States; 8 Koneksa Health New York, NY United States; 9 Sage Bionetworks Seattle United States; 10 University of California, San Francisco San Francisco United States; 11 University of Pennsylvania Philadelphia United States

**Keywords:** Parkinson disease, falls, decentralized, digital health technologies

## Abstract

**Background:**

Falls and cognitive impairment are major sources of disability in Parkinson disease (PD). The ability to accurately identify individuals with PD at high risk for falls and cognitive impairment would provide an opportunity for intervention and potentially improve long-term outcomes. In a previous study, Assessing Telehealth Outcomes in Multiyear Extensions of Parkinson Disease Trials (AT-HOME PD), we remotely characterized participants with early PD who had participated in 1 of 2 PD clinical trials over 2 years of follow-up. These participants with advancing disease provide a unique opportunity to examine whether the capture of objective in-home measures via digital tools and bothersome symptoms via direct participant report improves the prediction of disease milestones.

**Objective:**

Assessing Telehealth Outcomes in Multiyear Extensions of Parkinson Disease Trials–2 (AT-HOME PD2) aims to examine whether digital tools and remote participant reporting can improve the prediction of falls and cognitive impairment, quantify changes in physical activity over time, and explore the relationship between physical activity and clinical progression over time.

**Methods:**

This is a decentralized observational study of up to 200 individuals with PD, with clinical and digital phenotyping for up to 3 years of follow-up. Participants are those who took part in the STEADY-PD III (NCT02168842), Study of Urate Elevation in Parkinson’s Disease, Phase 3 (SURE-PD3; NCT02642393), AT-HOME PD (NCT03538262), or PD GENEration (NCT04057794) studies. All participants complete 2 video visits per year, wear 2 wrist-worn sensors (Fitbit Charge 5 and ActiGraph CentrePoint Insight Watch) for 1 week each month, complete smartphone-based motor tasks (using the mPower 2.0 app) for 10 days each quarter, and complete online surveys (within the companion Fox Insight study) each quarter. Falls are assessed via a weekly automated telephone call. A cognitive diagnosis is determined by a consensus committee that considers scores on a global cognitive measure, detailed neuropsychological tests, a cognitive-related disability measure, and clinical information. Prediction models will be constructed, and prediction accuracy will be compared across the models.

**Results:**

Recruitment for the study was initiated in September 2023. Enrollment is ongoing, with 142 participants enrolled as of January 2025. Within the cohort, the average age is 69.2 (SD 8.7) years; 85 (59.9%) participants are male, 137 (96.5%) are White, and 2 (1.4%) are Hispanic or Latino; and the average disease duration is 8.9 (SD 1.3) years.

**Conclusions:**

AT-HOME PD2 is remotely clinically and digitally phenotyping participants with midstage PD to predict falls and cognitive impairment and to provide insights into long-term progression.

**International Registered Report Identifier (IRRID):**

DERR1-10.2196/71955

## Introduction

Parkinson disease (PD) is a progressive, neurodegenerative disorder that is increasing in prevalence [1]. Falls and cognitive impairment are major sources of disability in PD, associated with loss of independence, hospitalization, long-term care facility placement, and mortality [2-7], contributing to the estimated US $52 billion annual cost for PD care in the United States [8]. The early identification of individuals at risk for falls and cognitive decline is critical to enable appropriate intervention, which may take the form of home modifications, medication changes, or other adjustments, and to improve long-term outcomes. While many PD-specific and nonspecific risk factors for falls have been identified, the single best predictor for falls remains a prior history of falls, and new, more accurate models for fall prediction are needed [9-11]. Similarly, while many PD-specific and nonspecific risk factors for dementia have been identified [12-17], few studies have examined predictors of the new onset of PD-related mild cognitive impairment [18-20].

Digital tools, which enable frequent and objective in-home assessment of motor [21-24] and nonmotor function [25-29], stand to improve the management of PD and may improve the prediction of falls and cognitive impairment. However, they are not routinely used in clinical practice to aid in the prediction of falls or cognitive impairment. Many fall prediction models incorporate performance-based tests, which are typically conducted in a traditional clinic setting. However, relevant metrics, such as gait speed, cadence, and stride time variability, can be measured with digital tools in the home [11,30,31]. Studies have demonstrated the potential to use digital tools to predict falls and time to a first fall [32,33]. Digital tools have also been used to demonstrate that slower gait speed, lower levels of physical activity, less time spent outside of the home, and fewer trips outside the home are associated with cognitive impairment in older adults [34,35]. Novel methods of capturing the most bothersome symptoms and their functional impact [36] can generate a participant-reported natural history of PD [37] that may contextualize and inform understanding of data obtained from objective in-home assessment. Studies are needed to determine whether in-home digital metrics and remote participant reporting can improve the prediction of important PD-related clinical milestones.

In a previous study, AT-HOME PD (Assessing Telehealth Outcomes in Multiyear Extensions of Parkinson Disease Trials), we remotely characterized motor and nonmotor progression of participants with early PD using annual video visits, quarterly smartphone-based motor assessments, and quarterly online surveys over 2 years of follow-up [38]. AT-HOME PD was designed to assess a novel framework for the remote follow-up of clinical trial participants, to compare digital and traditional outcomes, and to identify novel digital disease measures. AT-HOME PD enrolled 226 participants (mean age 64.7, SD 8.8 y; 40% female; mean time since diagnosis 3.8, SD 1.2 y) from 42 US states and 1 Canadian province, who participated in either STEADY-PD III (NCT02168842) or Study of Urate Elevation in Parkinson’s Disease, Phase 3 (SURE-PD3; NCT02642393)—both multicenter, randomized, controlled, phase III studies of potential disease-slowing agents [38-40].

In this study, we will leverage this well-characterized cohort and describe the protocol for AT-HOME PD2 (Assessing Telehealth Outcomes in Multiyear Extensions of Parkinson Disease Trials–2), a decentralized observational study with clinical and digital phenotyping that will: (1) evaluate the extent to which digital tools and remote participant reporting can improve the prediction of falls and cognitive impairment compared with traditional models; (2) quantify change in physical activity and gait over time; and (3) explore the relationship between physical activity, development of falls or cognitive impairment, and symptom progression over time. We hypothesize that digital assessment of gait, sleep, and physical activity in the real world will improve the prediction of falls and cognitive impairment compared with a model composed of traditional measures.

## Methods

**Figure 1:**
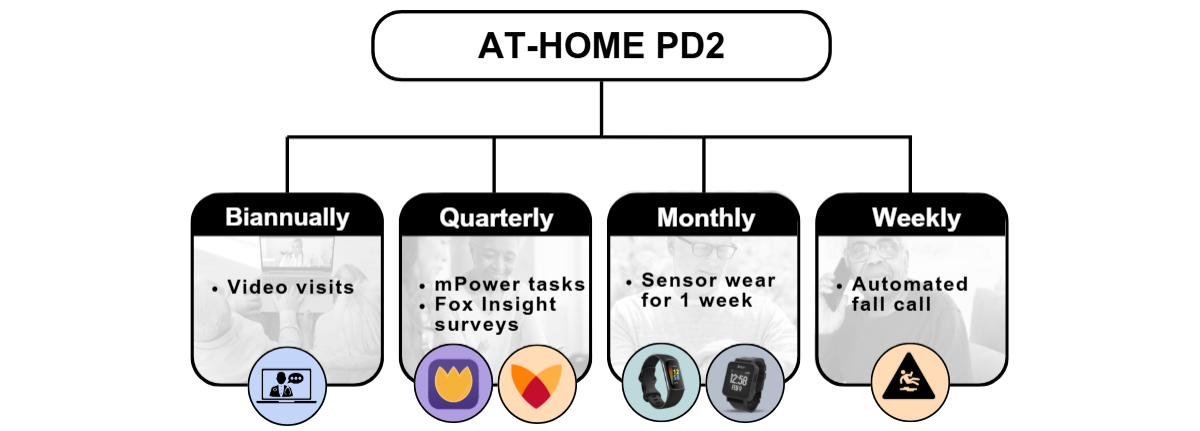
Overview of AT-HOME PD2 (Assessing Telehealth Outcomes in Multiyear Extensions of Parkinson Disease Trials–2) study design.

AT-HOME PD2 is a decentralized, observational PD study with up to 3 years of follow-up. All visits and assessments are conducted remotely in the participant’s natural environment. Participants complete 2 video visits per year, wear 2 sensors (Fitbit Charge 5 and ActiGraph CentrePoint Insight Watch) for 1 week each month, complete smartphone-based motor tasks (using the mPower 2.0 app) each quarter, and complete online surveys (via Fox Insight) each quarter (Figure 1).

### Study Setting

Visits are conducted by video at the University of Rochester using a cloud-based, HIPAA (Health Insurance Portability and Accountability Act)–compliant platform.

### Ethical Considerations

This study was approved by the University of Rochester Institutional Review Board (STUDY00008433). All participants provide informed consent and may choose to opt out of the study. Participants receive US $50 for each video visit, for up to a total of US $350, and keep the Fitbit upon completion of the study. The study team makes every effort to maintain data confidentiality and protect participants’ personal information. This includes assigning each participant a globally unique identifier, which is used to label collected information; restricting access to the study databases; transferring study data securely; and deidentifying study data before sharing it with qualified external researchers.

### Study Participants

Eligible participants were originally defined to be those who participated in AT-HOME PD and those who participated in STEADY-PD III (NCT02168842) or SURE-PD3 (NCT02642393) and provided consent to future research contact but did not participate in AT-HOME PD. Participants must have access to an internet-enabled device that supports video visits. Participants are provided with a webcam, Bluetooth adapter, smartphone stand, tablet stand, or an Android study phone with a data plan, when needed, to enable participation. There are no other technological requirements. A history of prior falls or cognitive impairment does not exclude individuals from participating.

In December 2024, because of concerns regarding our ability to meet the enrollment target, recruitment was opened to individuals who participated in PD GENEration (NCT04057794), a PD genetic testing study led by the Parkinson’s Foundation [41,42]. Specifically, AT-HOME PD2 participation was opened to individuals who participated in the first 2 phases of PD GENEration, which included clinical assessments [42]. The eligibility criteria are the same, with 2 exceptions. Participants from PD GENEration must have been diagnosed with PD 6 to 12 years ago and possess an iPhone. The disease duration requirement was added to ensure that newly recruited PD GENEration participants had a similar disease duration to those already participating in AT-HOME PD2. The iPhone stipulation was added, given the plans to incorporate the use of a smartphone app that is available only on iOS systems.

### Study Procedures

#### Enrollment of the STEADY-PD III, SURE-PD3, and AT-HOME PD Participants

Outreach to potential participants is conducted via telephone, email, and mail. Interested potential participants are sent a link to the electronic informed consent (eConsent) and scheduled for a screening visit, which may be performed by telephone or video. During the screening visit, informed consent is obtained, eligibility is confirmed, demographics are collected, and the need for equipment is assessed. The Fitbit Charge 5, ActiGraph CentrePoint Insight Watch, and any other needed equipment are shipped to the participant after the screening visit. Enrollment occurs on a rolling basis, with the enrollment visit scheduled within 60 days of the screening visit, and participants from the original AT-HOME PD study joining between month 48 and month 72, relative to their baseline AT-HOME PD visit. During the enrollment visit, participants receive instructions on mPower 2.0, Fitbit Charge 5, and ActiGraph CentrePoint Insight Watch use and, if required, assistance in enrolling in Fox Insight. All other assessments are as described subsequently and listed in Table 1.

**Table 1:** The assessments conducted during annual video visits.

Assessment domain	Instrument or measure
Motor assessments	Movement Disorder Society–Unified Parkinson’s Disease Rating Scale parts II, III, and IV Clinician falls and freezing assessment Patient falls assessment mPower 2.0 motor tasks
Cognitive assessments	Montreal Cognitive Assessment CANTAB^a^ connect: Rapid Visual Information Processing CANTAB connect: Paired Associates Learning CANTAB connect: Pattern Recognition Memory CANTAB connect: Spatial Working Memory CANTAB connect: One Touch Stockings of Cambridge
Other nonmotor assessments	Movement Disorder Society–Non-Motor Scale Movement Disorder Society–Unified Parkinson’s Disease Rating Scale part I
Global assessments	Schwab and England Activities of Daily Living Clinical Global Impression (Severity and Change)^b^ Patient Global Impression (Severity and Change)^b^
Study assessments	System Usability Scale Perceived Research Burden Assessment

^a^CANTAB: Cambridge Neuropsychological Test Automated Battery.

^b^Clinical Global Impression–Change and Patient Global Impression–Change are only completed during the follow-up visits.

#### Enrollment of PD GENEration Participants

The Parkinson’s Foundation sends PD GENEration participants an email invitation to participate in AT-HOME PD2. Interested potential participants who provide their consent to be contacted by the AT-HOME PD2 study team will then complete a screening form. Outreach to potential participants is conducted via telephone, email, or mail. The remainder of the procedures is as described earlier.

#### Video Visits

Participants are followed for up to 3 years and complete annual video visits with a comprehensive clinical assessment and interim video visits to support adherence to study activities. Patient-reported outcomes are completed within Research Electronic Data Capture (REDCap) outside of the scheduled clinical assessment visit. Some outcomes—falls assessment, Movement Disorder Society–Unified Parkinson’s Disease Rating Scale (MDS-UPDRS) part Ib and II [43], and Patient Global Impression-Severity and Change [44]—are completed before the video visit, while others—System Usability Scale [45] and Perceived Research Burden Assessment [46]—are completed after the video visit. The remainder of the assessments (Table 1) and any incomplete pre–video visit patient-reported outcomes are completed during the video visit. Participants also complete up to 3 interim research coordinator–led video visits. These are brief visits during which the mPower 2.0 active tasks are completed and adherence with the digital health technologies is reviewed.

#### Remote Monitoring With Digital Health Technologies

Within the mPower 2.0 smartphone app, participants complete 3 active motor tasks (ie, resting tremor, gait and balance, and finger tapping) each day for 10 days every 3 months. To minimize variability in performance, participants are instructed to perform the tasks during their self-identified PD medication ON state and to complete them at approximately the same time each day. Participants are also asked to complete 3 cognitive tasks (ie, dimensional card sort, digit symbol substitution test, and spatial working memory) once every 3 months. Participants may optionally consent to passive monitoring of movement and motor activity patterns.

Participants wear 2 sensors, the Fitbit Charge 5 and the ActiGraph CentrePoint Insight Watch, on their nondominant wrist for 7 days each month. Specifically, participants are instructed to wear the sensors during waking hours for 6 days and one complete 24-hour period on the seventh day. Participants are randomly assigned to wear either the Fitbit Charge 5 or the ActiGraph CentrePoint Insight Watch in the most distal position. Participants use the Fitbit app and the CentrePoint Connect app to sync their data to the Fitabase and CentrePoint platforms, respectively, throughout their wear week. The Fitbit Charge 5 is equipped with an accelerometer and photoplethysmography sensor. It captures wear time, motor parameters (eg, steps taken, distance, active zone min), and nonmotor parameters (eg, heart rate, heart rate variability, breathing rate, skin temperature, oxygen saturation, sleep stages, and sleep interruptions). The ActiGraph CentrePoint Insight Watch is equipped with an accelerometer and a near-body temperature sensor. It captures wear time, motor parameters (eg, steps taken, nonsedentary time, and physical activity), and nonmotor parameters (eg, sleep time, sleep onset latency, and sleep efficiency).

Adherence to digital health technologies is assessed during each visit. Participants receive a monthly calendar that indicates expected sensor wear and mPower 2.0 use. During the review of the calendar with the participant at the enrollment visit, the coordinator encourages them to inform the team if they need to adjust or reschedule a month’s sensor wear week to accommodate their personal schedule. Outside of the scheduled visits, the study team sends a reminder to the participant shortly before their next sensor wear week. The study team conducts monthly monitoring of the CentrePoint and Fitabase dashboards to monitor sensor battery levels, ensure consistent data uploads are completed by the participant, and identify any errors that require troubleshooting. If the battery level of a sensor falls below 20% or a data upload error occurs for a participant, the study team contacts the participant to assist with troubleshooting.

#### Fox Insight

Fox Insight is an online, observational research study sponsored by the Michael J Fox Foundation for Parkinson’s Research [47]. Every 3 months, participants complete a set of questionnaires, including the Patient Global Impression–Change, PD Patient Report of Problems [36], PD questionnaire-8 [48], and the Non-Motor Symptoms Questionnaire [49]. The Penn Parkinson’s Daily Activities Questionnaire [50], EQ-5D [51], and MDS-UPDRS part II are completed on a biannual basis. The Geriatric Depression Scale-15 [52], REM Sleep Behavior Disorder Single-Question Screen [53], and Physical Activity Scale for the Elderly [54] are completed annually.

#### Outcomes: Falls Assessment

Participants are provided with the following definition of a fall, based on an established expert consensus definition [55]: “a fall is an unexpected event in which you come to rest on the ground, floor, or a lower level.” To limit the possibility of recall bias, participants receive a weekly automated telephone call during which they are asked to enter the number of falls they have had (if any) in the past week [56]. Participants who are unable to answer the automated telephone calls (eg, those on Canadian phone plans who cannot receive calls from the United States) receive a weekly email from the study team instead. Participants who report a fall receive follow-up from a study coordinator to gather additional details on the fall circumstances and outcomes.

#### Outcomes: Cognitive Assessment

A consensus committee comprised of movement disorder and cognitive experts will use cognitive, functional, and clinical data to assign a cognitive diagnosis (ie, normal cognition, mild cognitive impairment, or dementia) for each participant at each annual visit. The committee will consider scores—including change from baseline—on a global cognitive measure (Montreal Cognitive Assessment) [57,58]; on specific neuropsychological tests (ie, Paired Associates Learning, Pattern Recognition Memory, Spatial Working Memory, One Touch Stockings of Cambridge; and Rapid Visual Information Processing from the Cambridge Neuropsychological Test Automated Battery) [59], and on the 15-item Penn Daily Activities Questionnaire, a measure of cognition-related functional abilities. The committee will also consider clinical information, such as the use of cholinesterase inhibitors.

### Safety Monitoring

We capture information on events of interest (eg, hospitalization, participation in a clinical trial, and neurological events) as spontaneously reported by the participant or observed by a member of the study team. To reduce the risk of falls during study assessments, participants who typically use an assistive device or have the assistance of another person to walk are instructed to complete walking tasks under their usual conditions. Participants and/or any member of the study team may opt not to complete walking tasks because of a perceived safety concern. We have a medical safety escalation plan that guides the response of the study team to medical or safety issues that may arise during a video visit. For example, in the event of an urgent medical concern that requires immediate attention, the participant or care partner is instructed to contact emergency services. If they are unable to do so, a member of the study team will contact emergency services on their behalf and remain in contact with the participant until emergency services arrive.

### Data Sharing

All study data from the video visits, mPower 2.0, Fitbit, ActiGraph, Fox Insight, and falls assessments will converge in Synapse (Sage Bionetworks), a cloud-based data management platform. Within Synapse, deidentified clinical and digital data will be available to qualified researchers. Clinical data will be transferred to the Parkinson’s Disease Biomarkers Program Data Management Resource, merged with data from the STEADY-PD III, SURE-PD3, AT-HOME PD, and PD GENEration parent studies, and made available to qualified researchers.

### Statistical Analysis

Statistical analysis will focus on (1) determining the extent to which digital tools and remote participant reporting improve the prediction of falls and cognitive impairment; (2) quantifying change in physical activity and gait metrics over time; and (3) exploring the relationship between physical activity, falls, cognitive impairment, and progression.

Prediction accuracy for time to first fall after baseline (AT-HOME PD2 enrollment), time to a recurrent fall (2 falls within 1 year), time to first-ever fall, and development of mild cognitive impairment and dementia at 1, 2, and 3 years after baseline will be compared among (1) a model using traditional measures; (2) an augmented model that also includes measures captured by remote assessment of motor and cognitive function; and (3) an augmented model that further includes participant-reported bothersome symptoms. Predictors included in the traditional model will comprise age; sex; disease duration (ie, years since diagnosis); measures of disease disability (Schwab and England Activities of Daily Living and Hoehn and Yahr); prior history of falls; prior history of freezing of gait; cognitive performance (Montreal Cognitive Assessment); medication use; measures of disease severity (MDS-UPDRS, MDS-Non-Motor Scale); and, for prediction of cognitive impairment, markers of socioeconomic status (ie, education and income level). The augmented digital model will also include digital measures of gait, physical activity, steps taken, heart rate, sleep parameters, motor function, and cognition. The augmented participant report model will also include curated descriptions of postural instability and cognition. Prediction models will be constructed using both traditional Cox proportional hazards regression and logistic regression, as well as survival or random forest classification methods. Prediction accuracy will be compared using the relative concordance index (for falls) or the area under the receiver operating characteristic curve (AUC; for mild cognitive impairment and dementia). We will evaluate the incremental value of the augmented models based on an improvement in the concordance index or AUC that is significantly greater than 0.05 at a 2-tailed significance level of *P*<.05.

We will calculate the mean steps per day and the mean time spent in moderate-to-vigorous physical activity for the Fitbit (using proprietary algorithms) and for the ActiGraph (using PD-specific cut points [60]) for each 7-day monitoring period. Gait dynamics (eg, step regularity and freezing of gait) will be extracted from active motor task performance and passively collected data within the mPower 2.0 app. We will visualize the interdevice reliability between the Fitbit and ActiGraph for step count and moderate-to-vigorous physical activity using Bland-Altman plots and quantify it using intraclass correlation coefficients.

Linear mixed effects models will be used to analyze the trajectories over time in physical activity, steps taken, and gait measures collected passively with the Fitbit, ActiGraph, and mPower 2.0 app over the 3-year follow-up period. Pearson or Spearman correlations will be used to assess the relationships between changes in these passively collected measures and changes in patient-reported outcome measures at specific follow-up time points.

Cox proportional hazards regression models will be used to examine the association between physical activity (ie, moderate-to-vigorous physical activity during the initial 7-day monitoring period) and the development of clinically relevant milestones (ie, the time to a first-ever fall and a fall after baseline, the time to a recurrent fall, the time to mild cognitive impairment, and the time to dementia). Associations between the trajectories over time in physical activity and traditionally assessed motor and cognitive progression will be examined by jointly modeling physical activity and symptom responses using mixed models that account for the correlation between these responses [61].

### Sample Size

In 2 midstage PD cohorts, 30% to 32% of participants experienced at least 1 fall over a year [62,63]. Assuming a sample size of 160 participants, a fall rate of 30%, and an AUC of 0.70 using traditional predictors, the study will have 80% power to declare that prediction using remote assessment measures is significantly better than an AUC of 0.75 if the true AUC for this model is at least 0.85. The study will have 80% power to declare that prediction using these measures in combination with reports of participant experience is significantly better than an AUC of 0.85 if the true AUC for this model is at least 0.94. A sample size of 200 participants will account for a 20% dropout rate.

### Study Oversight

The steering committee is composed of the principal investigator (RS), the study biostatistician (PA), and individuals with wide-ranging expertise (AA, JB, SB, KB, RD, AE, EM, DN, DO, SS, MS, TS, CT, and DW). The steering committee meets quarterly and provides oversight of the study, including the review of safety data and study progress, as well as oversight of analysis and publication plans.

## Results

A notice of award from the National Institutes of Health was received on April 3, 2023 (Multimedia Appendix 1). The study was approved by the University of Rochester Institutional Review Board in August 2023. Study recruitment was initiated on September 15, 2023, and is ongoing. The original pool of potential participants included 215 individuals from the AT-HOME PD study and 256 individuals from the STEADY-PD III or SURE-PD3 studies who had provided consent for future contact but did not participate in the AT-HOME PD study. Participation was opened to individuals from the PD GENEration study on November 18, 2024. As of January 15, 2025, 142 participants have enrolled. Of these, the average age is 69.2 (SD 8.7) years, 85 (59.9%) are male, 137 (96.5%) are Caucasian, 2 (1.4%) are Hispanic or Latino, and participants have a mean disease duration of 8.9 (SD 1.3) years. Among enrolled participants, 110 (77%) participated in the AT-HOME PD study, 9 (6%) in STEADY-PD III only, 8 (6%) in SURE-PD3 only, and 15 (11%) were recruited from the PD GENEration study. Among enrolled participants, 135 reside in 34 US states and the District of Columbia, and 7 reside in 1 Canadian province. Enrollment is anticipated to be completed by April 2025.

## Discussion

AT-HOME PD2 has enrolled 71% of the target number of participants from 3 research cohorts and is expected to generate new insights into the prediction of falls and cognitive impairment, inform the understanding of the role of physical activity in the management of midstage PD, and extend the longitudinal characterization of early PD clinical trial participants to up to 10 years through a participant-friendly, decentralized framework. Remote clinical and digital phenotyping in AT-HOME PD2 will complement in-person observational cohort studies, such as the Parkinson’s Progression Markers Initiative [64] and the Oxford Parkinson’s Disease Centre Discovery cohort [65], and long-term follow-up studies of other clinical trial cohorts, such as Levodopa in Early PD [66], and will provide insights into PD progression in the current treatment era. The inclusion of participants from PD GENEration will enable comparison of progression between clinical trial and observational research cohorts.

In AT-HOME PD2, traditional clinical assessments are complemented by digital tools, including a smartphone-based app, a commercial wrist-worn sensor, and a research-grade wrist-worn sensor. Many larger-scale digital phenotyping efforts have focused on populations with early-stage, untreated PD [67-69]; however, digital tools also have the potential to support the development of new treatments for and the care of individuals with later-stage PD. In AT-HOME PD2, we focus on a population with later-stage disease and use a combination of active motor assessments and passive data collection to generate insights into PD motor impairment, gait, physical activity, steps taken, and sleep. Our primary aim is centered on improving the prediction of falls and cognitive impairment—2 patient-centered disease milestones that are highly relevant to individuals with later-stage disease. To this end, we will use rigorous methods to capture the occurrence of falls and cognitive impairment. Falls are captured via a weekly automated telephone call, which will limit the potential for recall bias. Cognitive diagnosis is determined by a consensus cognitive committee comprised of experienced physicians who will use cognitive, functional, and clinical data to determine cognitive status [70].

Nonetheless, we recognize that this approach has limitations; for example, we anticipate that adherence to weekly fall reporting will decline over the study period. In addition, we recognize that some participants will have already experienced a fall or developed cognitive impairment at the time of study entry. While we will primarily focus on the prediction of time to first fall and development of mild cognitive impairment and dementia after study entry, secondary analyses will evaluate the prediction of fall frequency, time to first fall requiring hospitalization, time to first fall resulting in injury, time to first-ever fall, time to a recurrent fall, time to development of mild cognitive impairment, and time to development of dementia. In addition, we acknowledge that we do not capture every potential risk factor—for example, objective assessment of autonomic function is limited to the capture of heart rate, and neither of the wrist-worn sensors captures blood pressure. We also acknowledge the potential for differential attrition, with participants with greater physical or cognitive impairment more likely to be lost to follow-up. To partially address this, we collect contact information for a secondary informant at baseline.

Beyond the prediction of clinically meaningful events, this study will yield 3-year longitudinal data on change in motor and nonmotor digital measures, which will inform understanding of progression in this understudied stage of PD. Increased physical activity in PD is likely associated with slower decline [71,72] and a lower risk of mortality [73]; yet, studies to date on longitudinal change in physical activity have largely been limited to early PD or relied on self-report [68,74]. We will use active motor tasks and passive monitoring to objectively measure physical activity, steps taken, and gait parameters in a midstage PD sample. In turn, we will identify measures that capture progression over up to 3 years and examine the relationship with change in traditional clinical measures (eg, the MDS-UPDRS), and with the occurrence of clinically meaningful events. An improved understanding of the impact of physical activity on clinical outcomes will inform the counseling of individuals with midstage PD.

In addition, we will compare the performance of the consumer-oriented Fitbit with that of the research-grade ActiGraph device. Digital devices and algorithms must be validated in the appropriate context of use, which in this case is the monitoring of midstage PD in the home environment. While commercial devices are widely available, easily accessible, and may be acceptable for at-home assessment of physical activity in PD, they require validation before integration into clinical care or clinical trials. In this study, participants will wear the Fitbit and ActiGraph simultaneously on the same wrist, which will enable comparisons between devices. We will compare the feasibility of remote wrist-worn sensor assessment through an evaluation of wear-time adherence. We will also examine interdevice reliability through a comparison of physical activity, step count, and sleep parameters. The identification of a consumer device that accurately and reliably captures activity and sleep in PD would provide a potentially scalable method for monitoring these variables as part of clinical care.

In conclusion, in AT-HOME PD2, we will remotely clinically and digitally phenotype 200 participants with midstage PD, with the goal of improving the prediction of falls and cognitive impairment, characterizing long-term changes in physical activity, and examining the relationship between physical activity and clinical progression.

## Appendix

Multimedia Appendix 1 Peer review report from CIDH - Clinical Informatics and Digital Health Study Section, Healthcare Delivery and Methodologies Integrated Review Group (National Institutes of Health, USA).

## Abbreviations

AT-HOME PD: Assessing Telehealth Outcomes in Multiyear Extensions of Parkinson Disease Trials
AT-HOME PD2: Assessing Telehealth Outcomes in Multiyear Extensions of Parkinson Disease Trials–2
AUC: area under the receiver operating characteristic curve
HIPAA: Health Insurance Portability and Accountability Act
MDS-UPDRS: Movement Disorder Society–Unified Parkinson’s Disease Rating Scale
PD: Parkinson disease
REDCap: Research Electronic Data Capture
SURE-PD3: Study of Urate Elevation in Parkinson’s Disease, Phase 3

## Data Availability

The datasets generated or analyzed during this study will be available to qualified researchers in the Sage Bionetworks Synapse repository [75]. The clinical dataset generated or analyzed during this study will be available to qualified researchers in the Parkinson’s Disease Biomarkers Program Data Management Resource [76].
