# Supplementary material for: Improving Prediction of Falls and Cognitive Impairment in Parkinson Disease: Protocol for a Decentralized Observational Study
Source: JMIR Res Protoc. 2025 Aug 27;14:e71955. doi: 10.2196/71955 (PMC12423612; doi:10.2196/71955)
Supplement: Multimedia Appendix 1 [file resprot_v14i1e71955_app1.pdf]

1R01NS126933-01A1 Schneider, Ruth

EARLY STAGE INVESTIGATOR
NEW INVESTIGATOR

RESUME AND SUMMARY OF DISCUSSION: This resubmitted renewal application proposes to extend the observation of a cohort of mid-stage Parkinson's Disease patients and add a wearable fitness tracker and wrist worn sensor to observe whether the additional data collected is useful for generating new predictors and measure of PD progression relative to traditional measures collected via patient reported outcomes and remote assessments. The review panel were very enthusiastic about the value of the proposed research and its significance for providing a rich source of data for understanding mid- and late-stage PD that fills an informational gap for this population. The review panel noted that the research would provide a foundational data source that would inform future studies on the subject. The review panel found the investigators to be very strong, with strong knowledge and expertise on the use of devices for this population, with an ESI and new principal investigator who has experience working on the subject on prior research grants. The characterization of physical activity in the context of mid-stage PD patient trajectories was viewed as a highly innovative contribution. The validation of the commercially available Fitbit device that enhanced the prospects for generalizability was also viewed as an innovation. The review panel found the application to be driven by a series of strengths in the approach, with only minor weakness. The panel highlighted the wide array of measures that will be captured, the supply of devices and smart phones to enhance participation by underserved populations, and the measure of cognitive function, which was viewed as a key precursor to fall risk. The panel did note their preference for seeing a better characterization and description of dysautonomia given its relevance to mid-stage PD. The environment was considered excellent, with extensive history of conducting PD trials and the needed resources to conduct the study. The panel also assessed that the response to prior review was robust. In sum, there was a consensus among the panel that the proposed research would have a high impact for documenting the progression of PD in populations and finding new digital markers for understanding the trajectory of PD beyond what is currently known in the field.

DESCRIPTION (provided by applicant): The COVID-19 pandemic has disrupted clinical research and highlighted the value of patient centered research methods that enable participation from the home and collection of data directly from participants. Such decentralized research studies that harness video visits, digital tools and participant reporting, can reach a large, geographically dispersed population of participants, increase the frequency and scope of evaluation, and reduce the burden of participation. Parkinson's disease, a clinically heterogeneous neurodegenerative disorder that causes progressive disability, is well suited to such a model. Traditional assessments are typically subjective, insensitive to change, and limited to episodic administration and therefore fail to capture the complexity of Parkinson's disease. AT-HOME PD, the largest on-going decentralized longitudinal observational Parkinson's disease study with digital tools, is remotely characterizing ~225 participants with Parkinson's disease from two NINDS-funded, phase 3 clinical trials, STEADY-PD III and SURE-PD3. These studies yielded cohorts with comprehensive clinical phenotyping, whole genome sequencing, and serial plasma collection. AT-HOME PD participants are being characterized through video visits, smartphone-based assessments, and an online survey platform. The cohort is now approaching mid-stage Parkinson's disease, presenting an opportunity to advance our understanding of this under-studied population, improve the prediction of clinically relevant disease milestones like falls and cognitive impairment, quantify physical activity, and identify sensitive remote disease measures. This project will extend the follow-up of this cohort by 3 years and expand digital phenotyping of participants, using smartphone-based assessments and two wrist-worn sensors. The aims of this project are to 1) evaluate the extent to which digital tools and remote participant reporting can improve the prediction of

clinically relevant disease milestones compared with traditional measures, 2) quantify longitudinal change in physical activity, steps taken, and gait in mid-stage Parkinson's disease in the real-world, and 3) explore the relationship between physical activity and clinical outcomes in mid-stage Parkinson's disease. We will generate a dataset with approximately 10 continuous years of data on PD progression that begins prior to use of dopaminergic medications and progresses to midstage Parkinson's disease and beyond. This rich dataset will accelerate therapeutic development by filling knowledge gaps in the mid-stage Parkinson's disease population, helping to optimize models for conducting patient-centered remote research, evaluating new methods for predicting disease outcomes, and evaluating remote outcome measures.

PUBLIC HEALTH RELEVANCE: Traditional clinical research studies that require travel to a research site for in-person assessments place undue burden on participants with Parkinson's disease, particularly as the disease advances, and fail to capture the range and complexity of Parkinson's disease. This project will extend the remote follow-up of two clinical trial cohorts using video visits, remote patient reporting, smartphones, and wrist-worn sensors. By addressing some of the limitations of traditional assessments and evaluating new methods for predicting events like falls and cognitive impairment, we will help to accelerate the development of new treatments for people with Parkinson's disease.

CRITIQUE 1

Significance: 2
Investigator(s): 2
Innovation: 3
Approach: 2
Environment: 2

Overall Impact:

- This is a resubmission of a previously reviewed application. The applicants propose a 3-year extension of AT-HOME PD which is a longitudinal observational Parkinson's disease study that uses digital tools to remotely characterize PD patients.
- This study will allow for more robust characterization and follow-up of PD patients through the mid-disease timeframe in geographically dispersed populations. Mid-stage and late-stage PD patients have mobility issues that could preclude in person assessments, so developing robust telehealth and digital tools may prove clinically useful. The study will accrue 10 years of data on PD progression from prior to use of dopaminergic medications through mid-stage PD and explore the impact of physical activity on outcomes.
- The applicants address prior criticisms of the grant
- Overall, the methodology is robust and the applicants have the necessary experience and environment to successfully execute this study
- My only minor-moderate weakness would be that there are no assessments of dysautonomia (e.g., COMPASS-31) or evaluations of HR variability or BP that could conceivably be done using digital devices. Because dysautonomia is a strong risk factor for falls, it would be important to incorporate metrics that assess this into the schedule of activities.

1. Significance:

Strengths

- This study will allow for more robust characterization and follow-up of PD patients through the mid-disease timeframe in geographically dispersed populations. Mid-stage and late stage PD patients have mobilitiy issues that could preclude in person assessments, so developing robust telehealth and digital tools may prove clinically useful. The study will accrue 10 years of data on PD progression from prior to use of dopaminergic medications through midstage PD and explore the impact of physical activity on outcomes.
- Since this is a continuation of a longitudinal study, there is pre-existing whole genome sequencing and biological data
- The study will provide real world data on risk factors (particularly physical activity risk factors) for falls, which are a major cause of morbidity and mortality in PD patients.

Weaknesses

- None noted by Reviewer

2. Investigator(s):

Strengths

- The PI is an early stage and new investigator who has been at Rochester since med school thru neurology fellowship in movement disorders. She has been PI for 2 different funded studies and has been an investigator for AT-HOME PD
- Current PIs for AT-HOME PD are included as co-investigators for project continuity

Weaknesses

- None noted by Reviewer

3. Innovation:

Strengths

- The applicants propose a virtual follow-up of mid-stage PD patients which has not heretofore been performed with characterization of physical activity.
- Includes a validation of commercially available Fitbit (for generalizability) with research-grade actigraph
- Incorporates PROs

Weaknesses

- None noted by Reviewer

4. Approach:

Strengths

- The battery of study wide measures (in table 6) are comprehensive and the team has standardized experience in administering these batteries via video conference.
- The study will supply actigraphy devices and smart phones when needed to ensure enrollment of underserved populations

- I like that there are questionnaires evaluating perceived research burden and usability scales for devices, since this will impact generalizability of any digital assessments (in terms of compliance)
- Determination of cognitive status follows PD society guidelines and multi-domain neuropscyh testing tools are used over time and administered by trained personnel
- Prelim data on adherence to digital assessments and an adherence plan is provided (also the actigraph detects wear time and if the device has been removed)
- Applicants require a study partner to ensure adherence to the study protocol as PD patients may develop progressive cognitive issues

Weaknesses

- The applicants mention characterizing dysautonomia in mid-stage PD but I don’t see any dysautonomia questionnaires or tools. Since dysautonomia will impact fall risk, this should be considered. Is there a way to incorporate device-based BP testing?

5. Environment:

Strengths

- Rochester is a robust research center with CTSI and center for health and technology
- They have conducted 79 PD trials over 33 years

Weaknesses

- None noted by Reviewer

Study Timeline:

Strengths

- None noted by Reviewer

Weaknesses

- None noted by Reviewer

Protections for Human Subjects:

Acceptable Risks and/or Adequate Protections

Inclusion Plans:

- Sex/Gender: Distribution justified scientifically
- Race/Ethnicity: Distribution justified scientifically
- For NIH-Defined Phase III trials, Plans for valid design and analysis:
- Inclusion/Exclusion Based on Age: Distribution justified scientifically

Vertebrate Animals:

Not Applicable (No Vertebrate Animals)

Biohazards:
Not Applicable (No Biohazards)

Resubmission:

- Prior criticisms adequately addressed

Resource Sharing Plans:
Acceptable

Authentication of Key Biological and/or Chemical Resources:
Not Applicable (No Relevant Resources)

Budget and Period of Support:
Recommend as Requested

CRITIQUE 2

Significance: 2
Investigator(s): 2
Innovation: 2
Approach: 2
Environment: 1

Overall Impact: This proposal addresses a very significant issue in the field of geriatrics and neurological diseases (Parkinson's disease (PD)). The overall significance is high because the research is likely to provide answer several important questions, including development of measures of disease progression tracking at home as well as generating knowledge about PD symptoms progression over time (~8 years). The project is innovative in terms of the domain: no previous studies generated a longitudinal observation of PD patient's disease progression. The study is somewhat innovative in terms of methods used to conduct disease progression assessments. The team is outstanding with lead PI being a new investigator mentored by experienced investigators who were PI's on previous iterations of this project. Environment is excellent and has all material supports needed for the study. The approach is strong: this study will advance the rigor of prior research by generating/refining tools necessary for PD disease progression tracking in patient's home. The study is well positioned to develop and evaluate remote predictors and measures of disease progression to accelerate therapeutic development for mid-stage PD. Study aims are well described, and methods are appropriate for the given study aims. I am very enthusiastic about this proposal and study team.

1. Significance:
Strengths

- The project addresses a significant problem in the field of geriatrics. Specifically, it will generate a much needed but missing knowledge about Parkinson's disease (PD) progression in large cohort of patients.
- Continuous tracking of a PD patient cohort over 8 years will generate important insights that will help understand disease progression and trajectories of deterioration.

Weaknesses

- None noted by Reviewer

2. Investigator(s):

Strengths

- The team is comprised of experts in remote assessments, digital tools, and clinical trials and is uniquely qualified to accomplish these aims.
- The team is led by an early-stage investigator and there is strong support by experienced investigators who launched the original studies.

Weaknesses

- None noted by Reviewer

3. Innovation:

Strengths

- The study is innovative in terms of the domain: no previous studies generated a longitudinal observation of PD patient's disease progression.
- The study is somewhat innovative in terms of methods used to conduct disease progression assessments.

Weaknesses

- None noted by Reviewer

4. Approach:

Strengths

- This study will advance the rigor of prior research by generating/refining tools necessary for PD disease progression tracking in patient's home.
- The study is well positioned to develop and evaluate remote predictors and measures of disease progression to accelerate therapeutic development for mid-stage PD.
- Study aims are well described, and methods are appropriate for the given study aims.

Weaknesses

- None noted by Reviewer

5. Environment:

Strengths

- Environment is excellent and has all material supports needed for the study.

Weaknesses

- None noted by Reviewer

Study Timeline:

Strengths

- Appropriate timeline

Weaknesses

- None noted by Reviewer

Protections for Human Subjects:
Acceptable Risks and/or Adequate Protections

Inclusion Plans:

- Sex/Gender: Distribution justified scientifically
- Race/Ethnicity: Distribution justified scientifically
- For NIH-Defined Phase III trials, Plans for valid design and analysis:
- Inclusion/Exclusion Based on Age: Distribution justified scientifically

Vertebrate Animals:
Not Applicable (No Vertebrate Animals)

Biohazards:
Not Applicable (No Biohazards)

Resubmission:

- The study addressed previous review comments and made several minor and major changes.

Resource Sharing Plans:
Acceptable

Authentication of Key Biological and/or Chemical Resources:
Not Applicable (No Relevant Resources)

Budget and Period of Support:
Recommend as Requested

CRITIQUE 3

Significance: 2
Investigator(s): 1
Innovation: 2
Approach: 3
Environment: 1

Overall Impact: The goal of this proposal is to identify persons with Parkinson's disease (PD) who are at risk of near-term decline, including mobility, falls and cognitive decline to allow clinicians to intervene prior to incidents. They will accomplish this through a remote monitoring system to assess mobility and cognition with a high temporal resolution, in a longitudinal sample of patients with mid-stage PD where fully remote researcher (and ultimately clinical monitoring) can increase participation. The impact from these analyses of this transitional stage of PD, where independence is slowly lost and the risk of injury from falls increases, has a reasonable potential to inform clinical thinking about the role of monitoring persons with PD between visits to the neurologist. The study has innovative aspects, including the longitudinal design including data before mid-stage PD and the temporal granularity of data collection. The investigators are well suited to conduct this research and have a successful collaborative history working on the cohort in the prior study period. The environment is very good and has already proven to be appropriate for this study in the prior study period. The approach is overall reasonably strong, starting with proven feasibility and a very well-defined data analysis plan with demonstrated adequate power. The minor to moderate weaknesses include the lack of a justification for the missing at random assumption, even as a first-order approximation, and the lack of generalizability on the uptake of the remote monitoring technology due to the longstanding relationship with the participants.

1. Significance:

Strengths

- The ability to conduct frequent assessments of motion and cognition in persons with PD may identify important disease progression in between neurologist visits, to ensure preventative measures are taken before incidents involving falls occur.
- Remote monitoring also helps provide greater access to PD healthcare for those with transportation insecurity or in underserved regional settings.

Weaknesses

- While the goal of predicting the rate of decline (or disease progression) seems worthwhile, the link to how this information would change current treatment plans was not established.
- The hypotheses in Aim 2 and 3 seems supported in the literature, but it is unclear how the longitudinal aspect of the data will make those hypotheses more convincing.

2. Investigator(s):

Strengths

- The research team has a history of collaborative work, importantly including the AT-HOME PD study where most of the sample for this study will be recruited.
- The PI is a neurologist with extensive expertise in PD research including use of digital health technologies, including experience with this cohort.

Weaknesses

- None noted by Reviewer

3. Innovation:

Strengths

- Longitudinal focus on mid-stage PD to detect possible intervention cues is novel considering this length of period and sample size.
- The frequent objective datapoints provide the opportunity to detect trends not easily observed previously.
- The longitudinal relationship between physical activity, disease progression and clinical markers is understudied in the literature.

Weaknesses

- Sensor data is not novel, and some applications such as predicting falls even in this context seems incremental without a clearer clinical application

4. Approach:

Strengths

- The data analysis plan is well thought out, with an adequate demonstration of power and clear delineation of how the models test the hypotheses.
- The initial sample was recruited previously and successfully retained, making recruitment success quite likely here.
- Remote research in this cohort was successful during the covid era, and this study logically builds on that foundation.
- Sage Synapse is a stable database system that will facilitate collaboration and resource sharing.

Weaknesses

- The missing at random assumption may not be justified as missing data is likely to be correlated with disease severity, and should therefore be treated as a variety of censoring, and likewise, some specific caregivers may be less willing, able, or consistent in their help applying the technology to the person with PD.
- The rate of successful adoption of these technologies may not generalize as the researchers have a long-standing relationship with the participants, though this is minor as the value of the longitudinal data lies in other research questions.

5. Environment:

Strengths

- The environments for the project are quite good and opportune considering its current support for the AT-HOME PD study where this sample will mostly derive.
- The addition of Grey Matter Technology to analyze the PD-PROP data is appropriate.
- Bionetworks wholly specializes in the full-stack support for their PD app.

Weaknesses

- None noted by Reviewer

Study Timeline:

Strengths

- None noted by Reviewer

Weaknesses

- None noted by Reviewer

Protections for Human Subjects:

Acceptable Risks and/or Adequate Protections

- Patients are adequately protected by their protocol.

Inclusion Plans:

- Sex/Gender: Distribution justified scientifically
- Race/Ethnicity: Distribution justified scientifically
- For NIH-Defined Phase III trials, Plans for valid design and analysis:
- Inclusion/Exclusion Based on Age: Distribution justified scientifically
    - As this is a continuation of a prior study, the sex/gender and race/ethnicity breakdown in inherited from the initial study, which is justified only in that longitudinal studies are hard to conduct and restarting is not justified given the previous investment. Age is justified as this is a study of a disease that affects the older population.

Vertebrate Animals:

Not Applicable (No Vertebrate Animals)

Biohazards:

Not Applicable (No Biohazards)

Resubmission:

- The investigators were responsive to the previous review.

Resource Sharing Plans:

Acceptable

Authentication of Key Biological and/or Chemical Resources:

Not Applicable (No Relevant Resources)

Budget and Period of Support:

Recommended budget modifications or possible overlap identified:

- The budget is reasonable for a study of this scope.

THE FOLLOWING SECTIONS WERE PREPARED BY THE SCIENTIFIC REVIEW OFFICER TO SUMMARIZE THE OUTCOME OF DISCUSSIONS OF THE REVIEW COMMITTEE, OR REVIEWERS' WRITTEN CRITIQUES, ON THE FOLLOWING ISSUES:

PROTECTION OF HUMAN SUBJECTS: ACCEPTABLE

INCLUSION OF WOMEN PLAN: ACCEPTABLE

INCLUSION OF MINORITIES PLAN: ACCEPTABLE

INCLUSION ACROSS THE LIFESPAN: ACCEPTABLE

COMMITTEE BUDGET RECOMMENDATIONS: The budget was recommended as requested.

Footnotes for 1 R01 NS126933-01A1; PI Name: Schneider, Ruth

NIH has modified its policy regarding the receipt of resubmissions (amended applications).See Guide Notice NOT-OD-18-197 at https://grants.nih.gov/grants/guide/notice-files/NOT-OD-18-197.html. The impact/priority score is calculated after discussion of an application by averaging the overall scores (1-9) given by all voting reviewers on the committee and multiplying by 10. The criterion scores are submitted prior to the meeting by the individual reviewers assigned to an application, and are not discussed specifically at the review meeting or calculated into the overall impact score. Some applications also receive a percentile ranking. For details on the review process, see http://grants.nih.gov/grants/peer_review_process.htm#scoring.
